# Parenting self-efficacy and maternal mental health: the implications of cultural considerations for family-focused practice in the U.S

**DOI:** 10.3389/fpsyt.2025.1704371

**Published:** 2025-11-04

**Authors:** Kyra Sanchez, Patrice Wiley, Joanne Nicholson, Kaitlin M. Brooks, Francine M. Seruya, Rebecca Trenz

**Affiliations:** ^1^ Occupational Therapy Program, Mercy University, Dobbs Ferry, NY, United States; ^2^ School of Social and Behavioral Sciences, Mercy University, Dobbs Ferry, NY, United States; ^3^ Heller School for Social Policy and Management, Brandeis University, Waltham, MA, United States; ^4^ The Barbara H. Hagan School of Nursing and Health Sciences, Molloy University, Rockville Centre, NY, United States; ^5^ School of Health and Natural Sciences, Mercy University, Dobbs Ferry, NY, United States

**Keywords:** family-focused practice, maternal mental health, culture, parenting self-efficacy, Hispanic/Latina mothers

## Abstract

The potential benefits of family-focused practice for parents with mental illness have received increasing attention in the last decade, given the significant relationship between parenting status and mental health. It is important to place the experience of motherhood in a cultural context, an approach that is likely to enhance the effectiveness of interventions, services, and supports for mothers and their families. In this Perspective article, we propose that the relationship between parenting self-efficacy and maternal mental health is moderated by a woman’s cultural lens (perceptions of motherhood) and cultural context (i.e., family support and community resources). We consider these concepts as they reflect the experiences of Hispanic/Latina women living in the US as an example, informed by a scoping review of the related literature. The literature underscores the notion that these may change over time or vary, given shifts in acculturation and enculturation, family circumstances and needs, and children’s characteristics and stage of development. Consideration of these key concepts suggests implications for family-focused practice approaches, and the importance of relevant research measures and methods to demonstrate effectiveness.

## 1 Introduction

The potential benefits of family-focused practice for parents with mental illness have received increasing attention in the last decade, given the significant relationship between parenting status and mental health (1–6). Most recently, recommendations have been made for enhancing diversity in studies of parent-child interaction and parental wellbeing (1, 7, 8). In particular, attention has been brought to our relative lack of understanding of the ways in which cultural beliefs and values shape perceptions of parenting (e.g., motherhood), experiences of mental health or illness, and participation in interventions, services, and supports (9–16). If we are to understand the ways in which parenting experiences relate to maternal mental health, then we must consider these relationships through a cultural lens, placing the experience of motherhood in a cultural context. Taking a cultural perspective is likely to enhance the effectiveness of interventions, services, and supports for mothers and their families (12).

The notion of “family” sits squarely in frameworks of family-focused practice (FFP), particularly when the person challenged by mental health problems is a parent (12, 17, 18). This suggests that mothers are best considered “holistically” within the context of their family relationships, taking the expectations, values, and beliefs of family members as well as mothers’ own perspectives into account. Families convey cultural beliefs and traditions regarding motherhood, mental health, illness, and healing (12). The FFP approach to services embraces assessment and goal setting; instrumental, emotional and social support; psychoeducation; and connection with communities, with liaison, advocacy and coordination as key functions (17). As FFP maintains that family and community factors shape intervention outcomes, culture and context must be considered to ensure interventions, services, and supports for mothers are relevant and meaningful (13).

**Figure 1:**
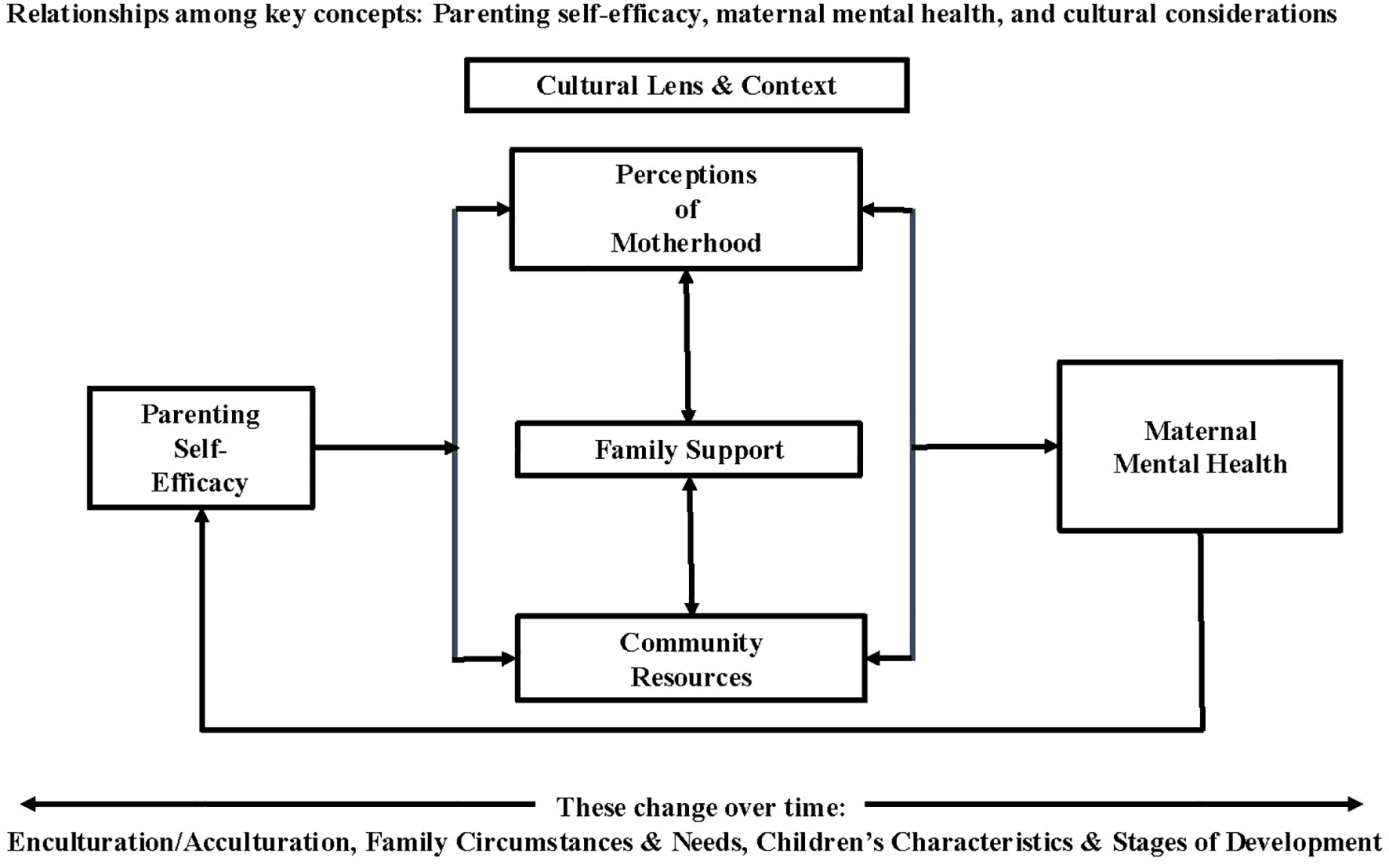
Relationships among key concepts: Parenting self-efficacy, maternal mental health, and cultural considerations.

The thesis proposed herein is that the relationship between parenting self-efficacy and maternal mental health (19) is moderated by a woman’s cultural lens (i.e., perceptions of motherhood) and cultural context (i.e., family support and community resources). (See **Figure 1**.) Our goal is to consider these concepts as they reflect the experiences of Hispanic/Latina women living in the US. We provide examples of the potential role of cultural variables as moderators in the relationship between parenting experience and maternal mental health, informed by a scoping review of the literature (20, 21). (See **Supplementary Material** for detailed scoping review procedures, PRISMA flow chart, and scoping review literature table.) The relationships among these variables may change over time or situation, depending on where mothers fall on the enculturation/acculturation continuum (22), family circumstances and needs (23–25), and children’s characteristics and stages of development (8, 22). Team discussions regarding the literature provided opportunity to minimize the influence of preconceived notions about Hispanic/Latina mothers and cultural concepts, and to reflect on subgroup variation. A culturally-informed understanding of the perspectives and experiences of mothers will enhance the development of relevant measures and methods and, ultimately, contribute to more effective family-focused interventions and practice, improving outcomes for all family members (13, 16).

## 2 Considering key concepts

Our goal is to consider *parenting self-efficacy* as a key concept reflecting parenting experience, seen through a cultural lens and context (i.e., *perceptions of motherhood*, *family support*, and *community resources*), as it relates to *maternal mental health*. The literature on the experiences of Hispanic/Latina women in the US provides insight into these concepts and relationships as well as underscores the notion that these may change over time or vary, given shifts in enculturation and acculturation, family circumstances and needs, and children’s characteristics and stage of development. Consideration of each of these key concepts suggests implications for family-focused practice approaches.

### 2.1 Maternal mental health

Experiences of motherhood and mental health are interconnected (22), and the mental health of parents can affect the whole family (12). Cultural influences may shape mothers’ perspectives on mental and reproductive health. As a case in point, Hispanic/Latina mothers are more likely to be diagnosed with anxiety or depressive disorder than their non-Hispanic/Latina counterparts, however, they may be less likely to pursue mental health treatment (19). Hispanic/Latina mothers who experience symptoms of postpartum depression remain dedicated to the family’s well-being, and may minimize their symptoms while fulfilling family obligations, possibly viewing their depression as something to be “silently endured” (26, 27).

Family and parenting stresses may contribute to or undermine maternal mental health (28). Hispanic/Latina mothers may be vulnerable to multiple adversities, including the stress associated with acculturation (i.e., assimilation to a different culture) which, in turn, may impact mental health negatively (19). Economic hardship, parent-child conflict, and acculturation-based family conflict may increase the risk of maternal depression (24) while access to material resources may lower the risk of mental health challenges (19). A higher level of parental stress, defined as the subjective feeling that results from the discrepancy between the perceived demands of parenting and the perceived ability to cope with those demands (19, 29), is related to increased maternal mental health risk in Hispanic/Latina mothers (19).

These compounding factors may affect how mothers experience and frame their mental health, and whether, how, and where they seek support. Their expression of challenges may revolve around child and family well-being, rather than personal acknowledgement of depression or anxiety. Providers must attend to “problems” as they are presented by mothers, rather than their diagnostic assessments of a mother’s mental status and set goals with mothers accordingly. For example, research has suggested the notion that conversations about stresses associated with motherhood, rather than about mental health per se, may increase the likelihood that Hispanic/Latina mothers engage in mental health services (19).

Hispanic/Latina mothers may be expected to be self-sacrificing and may focus on meeting the needs of others within their social sphere. An approach that highlights the potential benefit of an intervention for children and/or the entire family, consistent with a family-focused approach, may be more engaging to Hispanic/Latina mothers than a focus on addressing their own individual feelings and needs. To address the mental health of Hispanic/Latina mothers, service providers must be aware of these cultural factors to reframe needs assessment and goal-setting. and focus on delivery of care in ways that engage mothers and enlist the support of the whole family.

### 2.2 Parenting self-efficacy

The concept of parenting self-efficacy is often used in studying the parenting experience (30–33). Parenting self-efficacy (PSE) is defined as the confidence parents have in raising their children (30, 34), their perception of their parenting skills (35), and their belief in their ability to positively influence their children’s development (19). A mother’s level of PSE may impact her perception of herself as a mother and influence her parent-child relationship (30). When Hispanic/Latina mothers feel less confident in their ability to parent, they may be more likely to experience anxiety or depressive symptoms (19). As depression and parenting stress increase, mothers, in turn, may feel less confident in their ability to parent (30).

Cultural differences play a vital role in the attitudes, values, and beliefs a mother has of herself, which may influence her confidence in parenting (36). Hispanic/Latina mothers have higher levels of *cultural* parenting self-efficacy (PSE), defined as a parent’s ability to instill pride in their children by connecting them to the beliefs and values of their native culture, when compared with Asian parents (8). Hispanic/Latina mothers report higher cultural PSE when they are able to teach their children about their culture and help them gain an understanding and appreciation for their backgrounds (7, 8) while protecting them from what are perceived to be the “harmful influences” of American culture (37–39).

Cultural norms and parenting expectations may heavily influence childrearing practices; understanding these can inform approaches to family-focused practice with mothers and families (8, 13). If building parenting self-efficacy is an intervention focus, care must be taken to place this notion in cultural context, and assess outcomes accordingly. Intervention processes may need to be tailored to build on mothers’ strengths, as seen through their cultural lens and as perceived by family members (e.g., family elders) (25). Care must be taken to consider and respect families’ norms and expectations, while addressing mothers’ needs.

### 2.3 Perceptions of motherhood

Traditional Hispanic/Latina cultural beliefs and familial expectations suggest the stereotypic notion of the ideal mother as one who takes on multiple caregiving roles, childcare duties, and house management, while navigating the complex challenges of parenting (19, 25, 35, 38). This set of beliefs, framed as *Marianismo*, may shape how Hispanic/Latina women evaluate themselves as mothers (40, 41). A range of *Marianismo* beliefs has been described in the literature, from those women most traditional (i.e., who strongly align with culturally expected norms), to those who reject some expectations (e.g., submissiveness, not complaining) while endorsing others (e.g., being family-oriented), to those who hold contradicting beliefs (e.g., endorsement of virtue, but not spirituality) (41). While traditional gender roles (e.g., self-sacrificing, not complaining) may present challenges to wellbeing or contribute to psychological burden for Hispanic/Latina mothers, aspects of *Marianismo*, such as strong family ties, may provide essential support for mothers as they navigate parenting (26).

Hispanic/Latina women may show a selective and nuanced relationship with different aspects of *Marianismo* that suggest a diverse range of perceptions of the “perfect mother” (41). Although mothers may acknowledge that the concept of being a perfect mother is unattainable, they may believe mothers at their best are nurturing, communicative, cohesive, and responsive to the needs of their children (37, 38). Cultural values influence Hispanic/Latina mothers’ ideas of “successful parenting” (e.g., their child’s well-being and their own personal commitment to parenting) (25, 37). Hispanic/Latina mothers may especially face challenges in their perceptions of “perfect mother” and “successful parenting” when attempting to merge their cultural beliefs with beliefs of American culture (38).

A woman’s evaluation of herself as a mother, therefore, is likely closely tied to her psychological well-being and health. Mothers in the caregiver role have been found to have higher levels of life satisfaction when they perceive themselves as capable of effectively raising and educating their children (35). Overall, Hispanic/Latina mothers likely feel that their children are an important part of their self-identity and are worth sacrifices they have to make, which reflects their perception of themselves as mothers and the importance of their perceived success to their overall well-being (35, 38).

While not all Hispanic/Latina mothers fully endorse every aspect of *Marianismo*, those who align with self-sacrificing tenets may be deterred from seeking help, as vulnerability may be viewed as a sign of weakness. A family-focused practice approach emphasizes the importance of respecting mothers’ beliefs, rather than challenging them, to engage effectively (9). How well a woman is doing as a mother is pivotal to a family-focused approach, and may be an essential motivation for engaging in interventions and services for mothers with mental health challenges who want to perceive and experience themselves as capable. Addressing a mother’s needs may have positive impact on all family members (12).

### 2.4 Family support

Hispanic cultural beliefs related to the concept of *Familismo* emphasize placing family needs above individual needs, and highlight the importance of strong family support (19). While a Hispanic/Latina mother may sacrifice or suffer to meet the needs of family members, she may derive essential family support and help in navigating the challenges of parenthood, and be honored by her family for her sacrifice. A higher level of social support is a well-established predictor of optimal parenting practices and well-being (23). The extent to which Hispanic/Latina mothers receive support from their families has been found to be related to how efficacious mothers feel as parents (25). In this context, family support is found to be protective against depression (26, 28, 42).

Extended family may assist Hispanic/Latina mothers with child-rearing responsibilities. A grandmother’s emphasis on familism proves predictive of higher support of and communication with adolescent mothers as they navigate parenting (25). Support benefits mothers, particularly, if they are also given a sense of autonomy. Further, familial support and enhanced parenting self-efficacy are linked to improved outcomes for children (i.e., greater social competence in children at four years of age and greater academic functioning at 5 years of age) (25).

The health and well-being of Hispanic/Latina mothers depend on family dynamics and family relationships that recognize the challenges of motherhood, support a mother asking for help, and work to positively intervene, rather than criticize or undermine (26). Effective support involves the guidance and actions of family members that reduce a mother’s stress, while acknowledging a mother’s role and preference for autonomous decision-making. A family-focused practice approach may include the involvement of family members to understand their beliefs about parenting, mental health and healing, and the psychoeducation of family members to encourage their effective support of mothers with mental health challenges. Educating the entire family may help lessen stigma and encourage earlier identification of needs (26).

### 2.5 Community resources

Our focus, as we consider the implications of a cultural lens and context for family-focused practice, is on professional resources available in the community (e.g., mental health clinics, human service agencies). While Hispanic/Latina mothers may have access to family and neighborhood supports, they may face challenges in accessing professional mental health services (26, 37, 39, 43). Cultural messages and beliefs may serve as barriers that prevent them from acknowledging mental health conditions and seeking help from others outside the family (19, 26, 37, 39). Family members, especially those most closely tied to traditional cultural beliefs, may be unfamiliar with or unwilling to acknowledge mental health concerns. The anticipation of judgment by family members may prevent Hispanic/Latina women from help-seeking (26). Hispanic/Latina mothers may fear the risks that are associated with seeking services, such as disclosing private information related to their immigrant status (26). Additionally, the U.S healthcare system has limited availability of services and interventions that are tailored to meet the needs of Hispanic/Latina mothers (7, 8, 39, 42). Healthcare professionals may lack knowledge and resources that are relevant or meaningful to Hispanic/Latina mothers, limiting professionals’ ability to provide optimal services for this population (7, 8, 39, 42).

The barriers to accessing professional resources which Hispanic/Latina mothers may face have serious long-term implications for their mental health. The lack of tailored resources addressing cultural considerations and the needs unique to Hispanic/Latina women largely places these mothers in a position to be underserved. If Hispanic/Latina women feel judged for not conforming to the expectations of the ideal or perfect mother, they may avoid seeking help from their community and institutions. Mothers may be fearful of seeking treatment due to fear of negative outcomes, including deportation, further perpetuating stigma and avoidance of intervention services. Providers may not be informed or trained regarding cultural considerations (9). These factors may create obstacles that underscore the need for tailored, culturally relevant, family-centered practice approaches and culturally humble providers who effectively recognize and address the needs of Hispanic/Latina mothers and their families (44).

## 3 Discussion

This perspective article argues that the relationship between parenting self-efficacy and maternal mental health is moderated by a woman’s cultural lens and cultural context. We suggest that interventions, services, and supports for mothers must be family-focused to build on the strengths in women and families (e.g., leveraging family relationships and opportunities for support), capitalize on culturally-acceptable motivations (e.g., acknowledging the desire to be perceived as a capable mother), and non-judgmental (e.g., respecting mothers’ beliefs and goals) to optimize outcomes for both parents and children. It is our perspective that family-focused practice must take cultural considerations into account if interventions, services, and supports are to be most effective with the designated target population, in this example, Hispanic/Latina women and their families.

Hispanic/Latina mothers may be challenged in navigating the stages of childrearing and demands of motherhood while merging their cultural beliefs with American culture, depending on where they fall on the enculturation/acculturation continuum. Our perspective emphasizes the importance that family and community context have on shaping mother’s perceptions of themselves and their children, impacting their parenting self-efficacy and mental health. To support a woman during all stages of motherhood, it is important to consider the whole family and/or her support system while remaining culturally informed and sensitive. The development of culturally tailored parenting programs can support Hispanic/Latina mothers and their families. For example, interventions aimed at improving parenting self-efficacy, while aligning with Hispanic/Latina women’s identities and goals as mothers, and the values, norms, and expectations of family members, may be most effective. Intervention effectiveness may be increased when cultural relevance is emphasized (9, 14, 16).

A mother’s sense of parenting self-efficacy is enhanced when she observes positive developmental and/or behavioral outcomes in her child. Interestingly, interventions and measures of parenting self-efficacy per se may emphasize qualities or characteristics (e.g., level of assertiveness) that may be inconsistent with traditional cultural norms or expectations of Hispanic/Latina mothers. Programs should emphasize strength-based approaches that align to the mothers’ cultural values and beliefs while also introducing evidence-based strategies for parenting, implementing a whole family approach. As Hispanic/Latina mother’s cultural influences place their family’s well-being over their own, encouraging mothers to seek support as it relates to improving their overall family’s well-being may empower mothers to attend to their mental health needs. Framing the solution to challenges as addressing family-related problems or attending to children’s needs may be more effective than referring to situations as mothers’ mental health crises. An emphasis on supporting mental health as a protective factor instead of a limitation may help Hispanic/Latina mothers feel more confident and comfortable seeking services.

Community support plays a vital role and serves as a protective factor for Hispanic/Latina mothers. For example, enhancing access to tailored, relevant community-based services that provide culturally-acceptable parenting workshops or peer mentoring programs, offered in the language of choice, can be facilitated by responsive, respectful professionals to support Hispanic/Latina mothers. Mothers may prefer to connect with a network of like-minded mothers with similar backgrounds, and experiencing similar challenges, providing peer support. Access to tailored, effective, convenient, family-focused interventions, services, and supports may offer encouragement, reduce isolation, and empower mothers, improving outcomes for both themselves and their families. Clearly, attending to cultural considerations in developing or adapting, implementing, and testing interventions, services, and supports will contribute to effectiveness and inform strategies for replicating and reproducing interventions with new target populations, in new service contexts.

## Data Availability

The original contributions presented in the study are included in the article/**Supplementary Material**. Further inquiries can be directed to the corresponding author.
