# Supplementary material for: Parenting self-efficacy and maternal mental health: the implications of cultural considerations for family-focused practice in the U.S
Source: Front Psychiatry. 2025 Nov 4;16:1704371. doi: 10.3389/fpsyt.2025.1704371 (PMC12623373; doi:10.3389/fpsyt.2025.1704371)
Supplement: Supplementary file 1 [file DataSheet1.pdf]

Supplementary Material

Parenting Self-Efficacy, Maternal Mental Health, and Cultural Considerations: A Scoping Review Provides the Background

Kyra Sanchez, Patrice Wiley, Joanne Nicholson, Kaitlin M. Brooks, Francine M. Seruya, and Rebecca Trenz
Maternal Mental Health Research Training Center
Mercy University

October 2025

This Perspective piece was informed by a scoping review of the literature regarding Hispanic/Latina mothers, guided by the PRISMA statement (1). A scoping review was chosen as the relevant approach, as our goal was to clarify key concepts and relationships among them (2). A systematic search of 5 databases was conducted in January 2025: CINAHL complete, MEDLINE, PubMed, PsycINFO, and PsycArticles. The following search terms were used with the Boolean operators AND/OR: *Hispanic or Latino or Latina or Mexican or Central American or South American or Hispanics or Latin AND mothers or mother or motherhood or maternal AND self–efficacy AND culture or cultural or ethnicity or identity or values*. In addition, articles were hand-searched by the study team using Google Scholar and the review of reference lists provided by authors in published articles. The article inclusion criteria were as follows: (1) full text; (2) peer reviewed; (3) academic articles; and (4) published in 2010 or later. Ninety-six articles were identified through the systematic database search. Sixteen articles were found by the study team using the hand search method. After reviewing the titles, twenty-one duplicate articles were removed. Following an abstract review, sixty-nine articles were excluded because they were not relevant to parenting self-efficacy. The literature search yielded 20 articles after duplicates and articles deemed irrelevant were removed.

The authors refined article selection based on the following criteria: (1) participants were Hispanic/Latina mothers or were included in the context of a larger study with other mothers or parents; (2) mothers were parents of children 2 years of age or older; (3) studies related to parenting self-efficacy and/or mental health; and (4) studies were relevant to the cultural concepts of marianismo, acculturation, reproductive justice, or other cultural influences. Two authors independently reviewed the articles. The determination was made to include one longitudinal pregnancy cohort study, given its focus on mothers' cultural values and mental health, and one study of mothers with prior postpartum depression experiences regarding the influence of cultural expectations. Literature matrices were created to streamline the extraction of information regarding specific sections of articles, including theoretical framework, methods, and results. One article, initially included in the review, was unanimously excluded. After the articles were analyzed, the reviewers used the literature matrices to conduct a manual thematic analysis relating to parenting self-efficacy and mental health among Hispanic/Latina mothers. Relevant excerpts from the articles were highlighted and themes recorded for further analysis. To ensure credibility and trustworthiness, independent reviewers agreed on the relevance of articles and subsequent themes in routine review sessions with the team. Discrepancies were resolved through collaborative decision-making.

Correspondence: jnicholson@brandeis.edu

The authors acknowledge the potential biases resulting from different cultural backgrounds and perspectives on motherhood, parenting, and mental health. We remain aware of the need to approach articles with an open and critical lens to minimize the influence of preconceived notions about Hispanic/Latina mothers and cultural concepts. The inclusion of open dialogue and regular discussions throughout the review process strengthened the trustworthiness and credibility of the analysis.

References

1. Tricco AC, Lillie E, Zarin W, O'Brien KK, Colquhoun H, Levac D, et al. PRISMA extension for scoping reviews (PRISMA-ScR): Checklist and explanation. Ann Intern Med. 2018 Oct 2;169(7):467–73. https://doi.org/10.7326/M18-0850

2. Munn Z, Peters MDJ, Stern C, Tufanaru C, McArthur A, Aromataris E. Systematic review or scoping review? Guidance for authors when choosing between a systematic or scoping review approach. BMC Medical Research Methodology. 2018 Nov 19;18(1):143. https://doi.org/10.1186/s12874-018-0611-x

Funding: The authors declare that financial support was received for the research and/or publication of this material. The financial support for this research was provided by the Health Resources and Services Administration (HRSA) Maternal Health Research Network for MSIs (HRSA grant number 1UR6MC50348-01-00) to the Mercy University Maternal Mental Health Research Training Center (MMH-RTC) through a collaborative subaward with Molloy University. The contents of this material do not necessarily represent the policy of HRSA and you should not assume endorsement by the Federal Government.

Correspondence: jnicholson@brandeis.edu

Figure 1. Parenting self-efficacy, maternal mental health, and cultural considerations: Scoping review PRISMA diagram

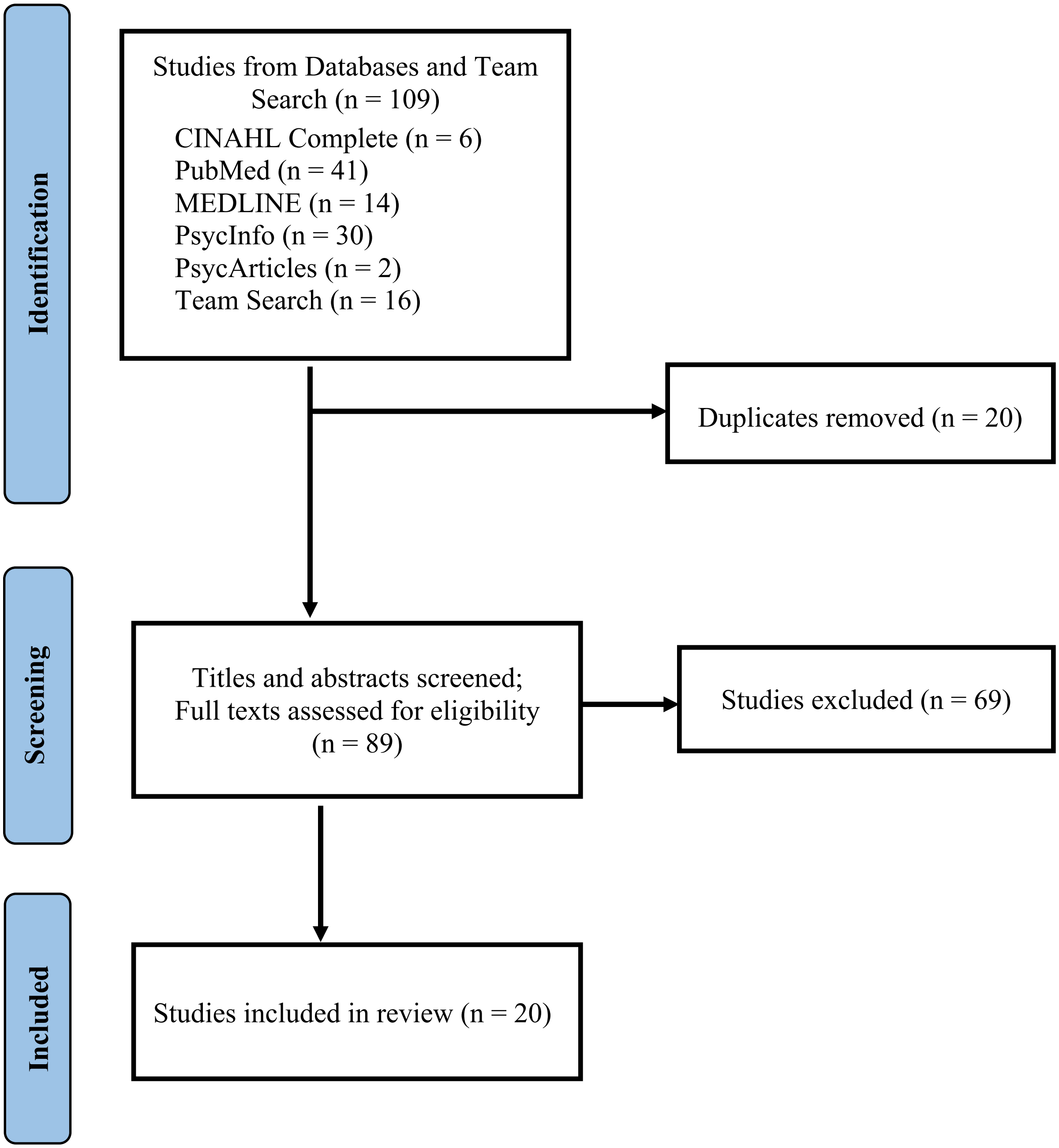

Correspondence: jnicholson@brandeis.edu

Table 1. Parenting self-efficacy, maternal mental health, and cultural considerations: A scoping review

Reference	Theoretical Framework	Methods	Results
(Bermudez et al., 2014)	Heuristic inquiry methodology<br><br>Feminist-informed intersectionality lens	Heuristic inquiry<br><br>Participants<br>20 mothers who identified as Hispanic, Mexican/Mexicana, Latina, Mexican American, Hispanic Vietnamese, low-middle class, Mean age = 31.7, Mean child's age = 7.6.<br><br>Measures<br><br>1- to 2-hour individual interviews (3 conducted in Spanish/18 in English<br><br>Interview data were coded and analyzed using heuristic inquiry	Motherhood was described by participants as a special, unique role that requires hard work and responsibility and is a personal growth experience.<br><br>Family and culture significantly influenced mothering beliefs and practices.<br><br>Disadvantages of being a Hispanic mother were mostly associated with experiences of discrimination.<br><br>Perceived differences in parenting were based on their perceptions of differences in discipline and family values.
(Boruszak-Kiziukiewicz & Kmita, 2020)	Bandura's Social Cognitive Theory	Systematic Review<br><br>Measures<br><br>Parental self-efficacy (PSE) - how well a parent believes they can positively influence their child's	Some studies show that parents who have successfully adapted to a majority culture show better psychological adjustment, higher levels of PSE, and positive parenting practices.

Correspondence: jnicholson@brandeis.edu

		behavior and development.<br><br>Different ways PSE was measured:<br><br>General - Overall beliefs on parenting self-efficacy<br><br>Domain General - Focus is on general confidence in being a parent. Not specific to parental tasks.<br><br>Domain Specific - conceptual beliefs on parenting self-efficacy as measured through a variety of parenting tasks (e.g., warmth, discipline)<br><br>Narrow Domain/Task Specific - confidence in managing a particular parenting task (e.g., toilet training).	Other studies show that as mothers become more acculturated, their parenting self-efficacy decreases.<br><br>Several interventions improved parenting self-efficacy and parent-child relationships. High completion rates and attendance prove that relevant interventions are needed.
(Calzada et al., 2019)	Family Stress Model<br><br>Acculturative stress - adjusting to a mainstream culture (learning the language, societal norms, and expected behaviors) while seeking to preserve cultural identity.	Statistical Analysis<br><br>Participants<br><br>175 Mexican-origin mothers with children 3 – 6 years of age living in a city in the Southwest	Mothers reported low levels of acculturative stress.<br><br>Nearly half of the sample showed clinical symptoms of depression.<br><br>Language differences and acculturative stress may make access to social support difficult, leaving mothers vulnerable to depression.

Correspondence: jnicholson@brandeis.edu

			Most mothers reported more authoritative parenting practices.<br><br>Children were twice as likely to have internalizing problems, such as anxiety and depression.
(Davis et al., 2015)	Culture influences Mexican-origin parents' environment and shapes their children's health behaviors.	Qualitative<br><br>Participants<br><br>39 Mexican-origin mothers participating in WIC (i.e., Special Supplemental Nutrition Program for Women, Infants, and Children), 18 years of age, with at least one child, aged 3 - 4 years, who consumed sugar-sweetened beverages or juice beyond recommended amounts.	Mothers wanted their child to be a good person (i.e., have good relationships with family and interact with people using kindness, love, respect, honesty, and humility).<br><br>Mothers wanted their children to be successful, where children were both educated and independent.<br><br>The perfect mother is responsive to their children's needs and helps them live fulfilling and happy lives.<br><br>Mothers wanted their children to understand and retain Mexican culture. This fostered a child's sense of belonging and encouraged closer familial relationships.

Correspondence: jnicholson@brandeis.edu

			Mothers used TV to encourage bilingualism (e.g., English, Spanish) while rejecting American foods, favoring a healthier Mexican diet.
(Dosanjh et al., 2023)	Developmental systems theory of resilience	Secondary Data Analysis<br><br>Participants<br><br>167 English-speaking Latina mothers (M =29) with at least one pre-kindergarten-aged child were recruited from 12 Title I public elementary schools.<br><br>Measures<br><br>Short Depression Scale, Parenting Stress Index-Short Form, Multidimensional Scale of Perceived Social Support (MSPSS), Covariates	Adverse childhood experiences (ACEs) are significantly associated with depression in Latina mothers.<br><br>Adverse childhood experiences (ACEs) do not directly cause parenting stress. However, ACEs can lead to depression and, as a result, can cause parenting stress.<br><br>High levels of family social support (e.g., family, friends) reduced the association between depression and parenting stress as it relates to ACEs.
(Driver & Amin., 2019)	Hispanic Health Paradox	Secondary Data Analysis<br><br>Participants<br><br>1096 Hispanic and non-Hispanic Black mothers from the Fragile Families and Child Wellbeing Study	Social support is associated with positive outcomes for minority populations, including reduced depression, improved mental health, and lower stress.<br><br>Higher levels of emotional

Correspondence: jnicholson@brandeis.edu

		(FFCWS)	support from partners significantly reduced parenting stress.<br><br>Hispanic mothers, both foreign- and native-born show lower levels of parenting stress than non-Hispanic Black mothers.<br><br>Mothers endorsing higher levels of marianismo experienced greater parenting stress.
(Dumka et al., 2010)	Social Cognitive Theory	Quantitative<br><br>Participants<br><br>189 Mexican American adolescents, in 7th grade, and their mothers.	Parenting self-efficacy predicted parents' positive control practices (e.g., monitoring behaviors, stable discipline) and adolescent conduct behaviors.
(Fang et al., 2021)	Process Model of Parenting	Systematic Review<br><br>Participants<br><br>Parents with children up to 18 years of age	Parenting self-efficacy is associated with parents' mental health, social support, relationship quality, and children’s behavior and temperament.<br><br>Mothers who were more depressed and experienced higher levels of parenting stress

Correspondence: jnicholson@brandeis.edu

			were less confident in their ability to parent.<br><br>Mothers who were satisfied with their role as a parent showed higher levels of parenting self-efficacy.<br><br>Culture affects the beliefs and attitudes about parenting self-efficacy.
(Fox, 2021)	Biocultural anthropological<br><br>Biopsychosocial Framework	Secondary Data Analysis<br><br>Participants<br><br>Wave 1 MCE study: 361 pregnant and postpartum women recruited from four sites in California<br><br>Wave 2 longitudinal pregnancy cohort study: criteria for participants were 18+, English or Spanish speaking, pregnant up to one year postpartum, self-identified as Latina, Hispanic, Chicana, Mexicana, and/or Latin American<br><br>Measures<br><br>Acculturation Rating Scale for Mexican Americans II (ARSMA-II), Edinburgh Postnatal Depression	The Latino acculturation paradox refers to the finding that first-generation Latino Americans tend to have better health than later generations, partly because acculturation can harm health by replacing protective traditional values with less healthy American behaviors and norms.<br><br>American cultural values system was positively correlated with depression and the Latino cultural values system was borderline correlated negatively with depression and positively with happiness.<br><br>Familism obligation was

Correspondence: jnicholson@brandeis.edu

		Scale (EPDS), Perceived Ethnic Discrimination Questionnaire Community Version [PEDQ-CV], Covariates: Socioeconomic status	associated with lower state anxiety.<br><br>More traditional gender roles were associated with greater perceived stress and lower happiness.
(Galvan et al., 2022)	Family Stress Model	Secondary Data Analysis<br><br>Participants<br>132 Latinx mothers,18 years or older, with a child enrolled or waitlisted in the Early Head Start Program.<br><br>Measures<br>Parental Self-Efficacy, Parenting Stress, Maternal Mental Health Risk, Maternal Perception of Material Resources	Mothers who felt less satisfied with their interactions with their child were more likely to experience maternal mental health risk (e.g., Generalized Anxiety and Depressive Symptomatology).<br><br>Among mothers with low parenting self-efficacy, those who perceived access to more material resources had fewer mental health challenges (i.e., lower mental health risk) than those who felt their resources were limited.<br><br>Risk of depression and anxiety decreased in mothers who were more enculturated (i.e., how closely someone aligns with their native heritage and culture).<br><br>Lower levels of parenting self-

Correspondence: jnicholson@brandeis.edu

			efficacy increased the risk of developing anxiety and depression.
(Harris et al., 2020)	Social-ecological perspective	Systematic Review<br><br>Measures:<br><br>Databases: PubMed, CINAHL, and PsycINFO.<br><br>15 out of 56 studies met inclusion criteria with a total of n= 5,656 mother-child participants	The mother's ability to perceive, interpret, and appropriately respond to a child's needs were more consistently correlated with maternal acculturation than maternal depression.<br><br>Economic stressors and lack of social support were significantly associated with maternal depression.
(Hill et al., 2019)	Family Stress Model	Secondary Data Analysis<br><br>Participants<br>169 adolescents and their mothers were recruited from two middle schools in semi-rural North Carolina	Economic hardship and poor parent-child relationships were associated with higher levels of maternal symptoms of depression.<br><br>Acculturation–based family conflict was predictive of maternal depression over foreigner-based discrimination or other stressors.<br><br>Latinas are more likely to be diagnosed with depression and anxiety (compared to their non-Hispanic counterparts) due to

Correspondence: jnicholson@brandeis.edu

			added stressors (i.e., financial hardship, acculturation, education), and are less likely to seek help.
(Kiang et al., 2017)	Cultural framework, Bandura's social cognitive theory	Qualitative<br><br>Participants<br><br>58 Asian American and 153 Latin American parents of children in grades 6–12<br><br>Methods<br><br>Acculturation conflict scale, Cultural Parental Self Efficacy (PSE) questions, Parenting Stress Index	Latino parents have less general parenting competence when both themselves and their children experience conflict with acculturation.<br><br>Latino American parents reported higher perceived self-efficacy in both heritage and American cultures compared to Asian American parents, providing them with the ability to more effectively communicate their cultural beliefs to their children while integrating mainstream American culture.
(Kiang et al., 2021)	Bandura's social cognitive theory, PSE theory, cultural and ecological models of childhood development	Quantitative<br><br>Participants<br><br>219 parents that identified as Latinx or Asian American (65% fathers; 35% mothers; 78% U.S.-born) of at least one child in grade 6–12	The more Latinx parents were involved in their child's life the greater perceived cultural parental self-efficacy they experienced.

Correspondence: jnicholson@brandeis.edu

		Measures<br><br>Cultural PSE, Parental involvement, child's grade, demographic variables	
(Matsuda et al., 2021)	Selye's stress theory	Secondary Data Analysis<br><br>Participants<br><br>172 mothers, the majority of whom were Hispanic/Latina, who received outpatient treatment for mental and substance use disorders	Mothers with mental disorders (e.g., mood/anxiety, psychosis, substance abuse) view being a mother as an important part of their identity.<br><br>Mothers want to improve their mental and physical health for their children, often prioritizing their needs.<br><br>Mothers reported feeling judged by family and health care providers, contributing to more emotional strain.<br><br>Mothers with a higher caregiving load (i.e., mothers who had multiple children, some of whom had health or psychological problems) were more fatigued, had higher BMI, and experienced pain.
(Mendez-Luck & Anthony, 2015)	Marianismo<br><br>Social Constructionist Framework	Qualitative Interviewing<br><br>Participants<br>44 Mexican-origin women, at least 18	Most participants viewed caregiving as a willing choice and commitment.

Correspondence: jnicholson@brandeis.edu

		years of age, are caregivers to a dependent elderly family member.	Perceptions of caregiving were affected by acculturative status. U.S.-born and native-born participants viewed obligation and duty differently.<br><br>U.S.-born participants viewed the term obligation negatively (and that it implied caregivers had no choice), while immigrant caregivers associated it with teachings about family values.<br><br>U.S.-born participants negatively viewed the term duty, expressing its part of the wifely role. For immigrant participants, duty was synonymous with love, gratitude for past contributions (i.e., intergenerational reciprocity), and honoring commitment.
(Palmer Rivera et al., 2024)	Marianismo Belief Scale	Narrative Literature Review<br><br>Methods<br><br>Database: APA PsychInfo, EBSCO Research Databases, JSTOR, and SAGE Journals.	Current marianismo research found positive and negative psychological consequences and outcomes in Latinas.<br><br>Women with strong family values and religious beliefs

Correspondence: jnicholson@brandeis.edu

		Inclusion criteria: articles that researched marianismo either quantitatively or qualitatively and were peer-reviewed.<br><br>Preliminary review: 20 articles were selected that met the criteria	tend to be more motivated in school, avoid substance use, show greater resilience to discrimination, and have strong support networks.<br><br>Women with strong marianismo beliefs are more likely to engage in unsafe sex, accept partner violence, and experience higher levels of depression.
(Royert et al., 2024)	Bandura's Social Cognitive Theory	Quantitative<br><br>Participants<br><br>700 women in charge of children less than 5 years of age from rural and urban areas of the Colombian Caribbean<br><br>Measures<br><br>Life satisfaction scale, Self-efficacy in childcare Likert scale, Scale of sexist attitudes	Mothers with higher parental self-efficacy reported lower life satisfaction influenced by various contextual factors (i.e., urban, rural, education level)<br><br>College-educated mothers with strong sexist beliefs about parenting felt more confident in their childcare abilities, which was linked to higher life satisfaction. This was connected to how they saw themselves in their

Correspondence: jnicholson@brandeis.edu

			caregiving role.<br><br>Social and cultural pressures in Hispanic mothers may affect mothers' parental self-efficacy and life satisfaction especially when related to gender expectations and taking on multiple caregiving responsibilities.
(Sampson et al., 2018)	Feminist Theory	Qualitative<br><br>Participants<br><br>19 Latina immigrant mothers from Mexico, Honduras, Nicaragua, and El Salvador with a prior positive postpartum depression (PPD) screen, average age 28, average household income of $23,000<br><br>Method<br><br>Three focus groups asking questions about PPD as it relates to cultural beliefs, family, and lived experiences.	Women's experiences with PPD were sadness, loneliness, confusion as they felt an overwhelming sense of responsibility for everyone else except for themselves.<br><br>Mothers expressed that their families' and communities' beliefs conveyed how new mothers should be able to handle responsibilities after childbirth.<br><br>Mothers believed that PPD is an American problem because in their native countries, sadness following childbirth, if it existed, was endured silently.

Correspondence: jnicholson@brandeis.edu

			Cultural expectations influence how mothers perceive PPD and its symptoms.
(Zeiders et al., 2015)	Belsky's Model of Parenting	Secondary Data Analysis<br><br>Participants<br>Mexican-origin adolescent mothers, between 15 and 18 years old, unmarried, within the third trimester of pregnancy, and had a mother figure.	Cultural values (e.g., familism) and norms influence perceptions of ideal parenting, parenting behaviors and methods, and social support networks.<br><br>Supportive behaviors from connected families help Latina adolescent mothers confidently parent.<br><br>Grandmothers who valued family were more likely to give parenting support and communicate with adolescent mothers over time.<br><br>Mothers felt more capable and confident as parents when supported by their grandmothers and given autonomy over their parenting decisions.

Correspondence: jnicholson@brandeis.edu

References

Bermúdez JM, Zak-Hunter LM, Stinson MA, Abrams BA. "I am not going to lose my kids to the streets": Meanings and experiences of motherhood among Mexican-origin women. Journal of Family Issues. 2014 Jan 1;35(1):3–27. https://doi.org/10.1177/0192513X12462680

Boruszak-Kiziukiewicz J, Kmita G. Parenting self-efficacy in immigrant families—A systematic review. Front Psychol [Internet]. 2020 May 26 [cited 2025 May 21];11. Available from: https://www.frontiersin.org/journals/psychology/articles/10.3389/fpsyg.2020.00985/fulhttps://doi.org/10.3389/fpsyg.2020.00985l

Calzada EJ, Sales A, O'Gara JL. Maternal depression and acculturative stress impacts on Mexican-origin children through authoritarian parenting. Journal of Applied Developmental Psychology. 2019 Jul 1;63:65–75. https://doi.org/10.1016/j.appdev.2019.05.001

Davis RE, Cole SM, Reyes LI, McKenney-Shubert SJ, Peterson KE. "It hurts a Latina when they tell us anything about our children": Implications of Mexican-origin mothers' maternal identities, aspirations, and attitudes about cultural transmission for childhood obesity prevention. Childhood Obesity. 2015 Oct;11(5):608–15. https://doi.org/10.1089/chi.2015.0011

Dosanjh LH, Vazquez C, Hess KE, McBride MJ, Kate Bearman S, Calzada EJ. Maternal depression and parenting stress in English-speaking Latina mothers with a history of adverse childhood experiences. Children and Youth Services Review. 2023 Jun 1;149:106889. https://doi.org/10.1016/j.childyouth.2023.106889

Driver N, Shafeek Amin N. Acculturation, social support, and maternal parenting stress among U.S. Hispanic mothers. J Child Fam Stud. 2019 May 1;28(5):1359–67. https://doi.org/10.1007/s10826-019-01351-6

Dumka LE, Gonzales NA, Wheeler LA, Millsap RE. Parenting self-efficacy and parenting practices over time in Mexican American families. Journal of Family Psychology. 2010;24(5):522–31. https://doi.org/10.1037/a0020833

Fang Y, Boelens M, Windhorst DA, Raat H, van Grieken A. Factors associated with parenting self-efficacy: A systematic review. Journal of Advanced Nursing. 2021;77(6):2641–61. https://doi.org/10.1111/jan.14767

Fox M. Discrimination as a moderator of the effects of acculturation and cultural values on mental health among pregnant and postpartum Latina women. American Anthropologist. 2021;123(4):780–804. https://doi.org/10.1111/aman.13665

Galvan T, Hurwich-Reiss E, Watamura SE. Latina mothers' mental health: An examination of its relation to parenting and material resources. Fam Process. 2022 Dec;61(4):1646–62. https://doi.org/10.1111/famp.12737

Correspondence: jnicholson@brandeis.edu

Harris RA, Jr HPS. Maternal depression in Latinas and child socioemotional development: A systematic review. PLOS ONE. 2020 Mar 12;15(3):e0230256. https://doi.org/10.1371/journal.pone.0230256

Hill DJ, Blanco Martinez S, Mejia Y, Kulish AL, Cavanaugh AM, Stein GL. Depressive symptoms in Latina mothers in an emerging immigrant community. Cultural Diversity & Ethnic Minority Psychology. 2019;25(3):397–402. https://doi.org/10.1037/cdp0000236

Kiang L, Glatz T, Buchanan CM. Acculturation conflict, cultural parenting self-efficacy, and perceived parenting competence in Asian American and Latino/a families. Fam Process. 2017 Dec;56(4):943–61. https://doi.org/10.1111/famp.12266

Kiang L, Glatz T, Buchanan CM. Developmental correlates of cultural parental self-efficacy among Asian and Latinx parents. J Child Fam Stud. 2021 Oct 1;30(10):2563–74. https://doi.org/10.1007/s10826-021-02065-4

Matsuda Y, McCabe BE, Behar-Zusman V. Mothering in the context of mental disorder: Effect of caregiving load on maternal health in a predominantly Hispanic sample. J Am Psychiatr Nurses Assoc. 2021 Sep 1;27(5):373–82. https://doi.org/10.1177/1078390320907693

Mendez-Luck CA, Anthony KP. Marianismo and caregiving role beliefs among U.S.-born and immigrant Mexican women. The Journals of Gerontology: Series B. 2016 Sep 1;71(5):926–35. https://doi.org/10.1093/geronb/gbv083

Palmer Rivera V, Colom Cruz A, Baco Bonilla G, Rivera Nieves Y, Nazario Ortiz A. The psychology of Marianismo: A review of empirical research. Salud y Conducta Humana. 2024;11(1). https://doi.org/10.71332/m10tyx05

Royert DR, Amar ,José Juan Amar, Romero Orozco ,Duban Rafael, and Palacio Sañudo JE. Relationship between sexist parenting, parental self-efficacy and life satisfaction in Colombian mothers: The moderating effect of gender-role-based parenting. Vulnerable Children and Youth Studies. 2024 Oct 1;19(4):695–707. https://doi.org/10.1080/17450128.2024.2404128

Sampson M, Torres MIM, Duron J, Davidson M. Latina immigrants' cultural beliefs about postpartum depression. Affilia. 2018 May 1;33(2):208–20. https://doi.org/10.1177/0886109917738745

Zeiders KH, Umaña-Taylor AJ, Jahromi LB, Updegraff KA. Grandmothers' familism values, adolescent mothers' parenting efficacy, and children's well-being. Journal of Family Psychology. 2015;29(4):624–34. https://doi.org/10.1037/fam0000103

Correspondence: jnicholson@brandeis.edu
